# Benthic Heterotrophic Protist Communities of the Southern Baltic Analyzed with the Help of Curated Metabarcoding Studies

**DOI:** 10.3390/biology12071010

**Published:** 2023-07-15

**Authors:** Maria Sachs, Manon Dünn, Hartmut Arndt

**Affiliations:** Institute of Zoology, General Ecology, Biocenter Cologne, University of Cologne, Zuelpicherstr. 47b, 51069 Cologne, Germany; msachs1@smail.uni-koeln.de (M.S.); manon.duenn@uni-koeln.de (M.D.)

**Keywords:** Baltic Sea, brackish, unicellular eukaryotes, diversity, amplicon sequencing, sediment

## Abstract

**Simple Summary:**

Unicellular eukaryotes (organisms with a nucleus), or protists, are an extremely diverse group of organisms and inhabit almost all environments. In the world’s oceans, they make up a large proportion of the overall diversity. Many heterotrophic protists feed on bacteria and, in this way, not only control bacterial abundance but also transport the bacterial-derived carbon to organisms at higher trophic levels in the food web. In recent years, many studies have focused on assessing the diversity of planktonic protists (organisms in the water column), but studies on seafloor dwelling (benthic) protists are much less frequent. So far, there are no extensive studies present that try to access the benthic protist communities in the Baltic Sea, one of Earth’s largest brackish water environments. Within our study, we try to make a first assessment of this diversity using the molecular technique of metabarcoding, which allows the simultaneous identification of many organisms from one sample via the barcoding of nucleic acids, such as DNA and RNA. To obtain an overview of how certain environmental factors such as salinity and water depth of the sediment may influence the community structure, we chose two regions of the southern Baltic.

**Abstract:**

Heterotrophic protists are key components of marine ecosystems. They act as controllers of bacterial and microphytobenthos production and contribute significantly to the carbon flux to higher trophic levels. Still, metabarcoding studies on benthic protist communities are much less frequent than for planktonic organisms. Especially in the Baltic Sea, representing the largest brackish water environment on earth, so far, no extensive metabarcoding studies have been conducted to assess the diversity of benthic protists in this unique and diverse habitat. This study aims to give first insights into the diversity of benthic protist communities in two different regions of the Baltic Sea, Fehmarnbelt, and Oderbank. Using amplicon sequencing of the 18S rDNA V9 region of over 100 individual sediment samples, we were able to show significant differences in the community composition between the two regions and to give insights into the vertical distribution of protists within the sediment (0–20 cm). The results indicate that the differences in community composition in the different regions might be explained by several abiotic factors such as salinity and water depth, but are also influenced by methodological aspects such as differences between DNA and RNA results.

## 1. Introduction

The marine benthal represents the largest habitat on earth, yet most studies focusing on the marine environment target the planktonic community. This is particularly detrimental for protist research, as benthic protist communities exhibit key ecosystem functions as main controllers of the bacterial and microphytobenthos production and the transfer of organic carbon to higher trophic levels. Moreover, marine sediments serve as seedbanks for planktonic communities [1] and, in this way, always comprise a mixture of actually active organisms, dormant stages, and free DNA [2]. Protists in general make up a large proportion of the molecular (and hence also functional) diversity in marine ecosystems [3]. This large diversity is naturally associated with a spread over several trophic levels. While phototrophic protists (e.g., diatoms and some dinoflagellates) may act as important contributors to primary production in shallow waters, heterotrophic protists, which form the focus of the present study, are voracious consumers of prokaryotes attached to particles in the sediment or being suspended in the pore water [4]. In addition, they act as decomposers for marine detritus [5], and as parasites [6,7]. The biology of heterotrophic protists comprises a large variety of lifestyles that have a strong influence on the marine carbon cycle through multiple food web connections [4].

Their adaptations to several trophic conditions allow them to exist in oxic as well as anoxic [8] environments and can make them indicators for certain environmental factors in the benthic realm.

The enclosed Baltic Sea represents one of the largest brackish water environments on Earth [9]. Through the inflow of saline water from the North Sea on the one hand and the inflow of freshwater from different rivers on the other hand, the Baltic Sea waters are stratified and offer a variety of salinity changes vertically and horizontally. The Baltic Sea is relatively shallow, with a mean depth of 60 m and—from a geological point of view—with 10,000 to 15,000 years being rather young [10] (its ecological age being approx. 8000 years [9]). The Baltic Sea has a large catchment area with heavy exploitation by humans, such as fisheries, pollution, and nutrient inflow via riverine runoff [10].

Within the Baltic Sea, several studies have focused on planktonic protist diversity, using “classic” cultivation methods [11,12] and clone libraries [13] within suboxic and anoxic waters [14], but also through metabarcoding in estuary regions [15] or along a salinity gradient [16]. Regarding benthic protists, some studies assessed diversity over live counting and staining [17,18,19] for the small-scale vertical distribution of heterotrophic protists in the sediment. Larger studies, estimating a broader benthic protist community (e.g., over metabarcoding), are so far missing for the Baltic Sea. Thus, the state of knowledge about benthic protists communities that are most likely shaped by the various abiotic conditions described above is poor with regard to the Baltic Sea. Previous studies have shown that especially grain size [20], as well as salinity [21], are important factors influencing benthic protist communities.

Earlier studies [22] usually tried to asses biodiversity through live counting of morphotypes, a task that not only requires a deep knowledge of the morphological characteristics of certain species, but can also be biased by the occurrence of cryptic species [23]. The large amount of metabarcoding studies in the past 10 years has contributed to resolving these issues and also unveiled several new protist lineages [24,25]. Nevertheless, classic methods have not lost their power. Classic taxonomical work gives sequences a “face” and an ecological meaning and is the backbone of public databases, without which an assignment and interpretation of the myriad of sequences produced through extended metabarcoding studies would simply not be possible.

Based on previous studies regarding benthic protist communities in littoral sites [22], we aimed to assess the diversity of benthic protist communities of the Baltic Sea through metabarcoding of the V9 region of the 18S rDNA in two sublittoral regions of the southern Baltic Sea.

## 2. Materials and Methods

### 2.1. Sampling

**Figure 1:**
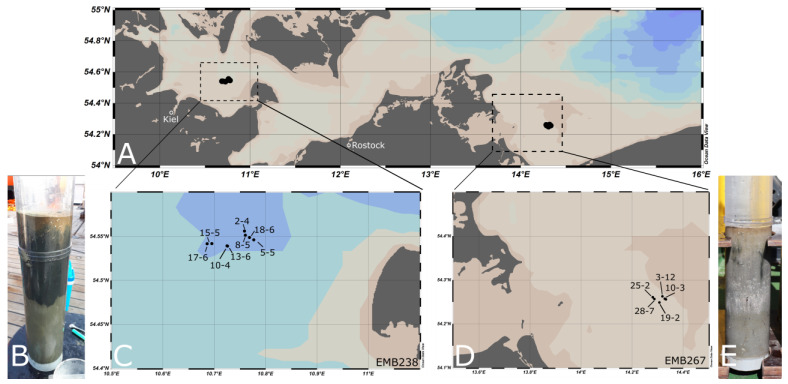
(**A**) Sampling regions of the two cruises in the western Baltic Sea, (**B**) MUC core taken from sediments of the Fehmarnbelt region, (**C**) close up of the sampling stations in the Fehmarnbelt region during cruise EMB238, (**D**) close up of the sampling stations in the Oderbank region during cruise EMB267, (**E**) MUC core taken from sediments of the Oderbank region. Maps were created using Ocean Data View [26].

The sampling for this study took place during two different cruises. The research vessel R/V Elisabeth Mann Borgese (EMB238) collected sediment samples from Fehmarnbelt at eight stations in 2020, four within the marine protected area (MPA) and four within a reference area. In 2021, sediment samples were taken at eight stations in the Oderbank region (EMB267), five stations from the MPA, and three from the reference area (Figure 1). For the metabarcoding studies, only a selection of samples was analyzed by the metabarcoding study (see Table 1). All sediment samples were taken with a Multicorer System (MUC).

For each station, three cores per station were taken and sliced into seven layers. If cores were too short, the interval was adjusted (Table 1). The chosen sampling regions significantly differed in environmental conditions. At Fehmarnbelt, the sediment was fine, dense, and muddy with a median grain size of around 55 µm, and the salinity at the bottom ranged around 19 PSU; in the Oderbank region, the sediment was much coarser and sandy with a median grain size of around 178 µm, and the salinity at the bottom ranged around 8 PSU.

### 2.2. DNA and RNA Extraction and cDNA Synthesis

For the Fehmarnbelt stations 17-6 and 18-6, we extracted DNA and RNA of each of the three cores, each with seven depth intervals by using the ZymoBIOMICS DNA/RNA Miniprep Kit (Zymo Research, Freiburg, Germany) using 250 mg of sediment per sample, as recommended for soil samples by the manufacturer. In principle, the kit allows a simultaneous extraction of DNA and RNA from the same sample. When RNA concentration after extraction was not sufficient for downstream processing using this kit, RNA was re-isolated using the RNeasy PowerSoil Total RNA^®^ Kit (Qiagen, Hilden, Germany) using 2 g of sediment. For the remaining stations, we extracted DNA from each of the three cores per station, but only for the upper two cm (0–1, 1–2 cmbsf = cm below seafloor), using the DNeasy Power Lyzer Power Soil^®^ DNA Isolation Kit (Qiagen, Hilden, Germany) to extract whole genomic DNA, but added additional pre-washing steps with three different washing solutions to improve downstream applications through removing potential contaminants [27,28] and adding further heating steps after bead beating [27].

For sediment samples from the Oderbank, we extracted only RNA using the RNeasy PowerSoil Total RNA^®^ Kit (Qiagen, Hilden, Germany). When RNA concentrations were too low after using 2 g of sediment per sample, we doubled the amount of sediment to 4 g, as recommended by the manufacturer.

For both sampling regions, RNA was synthesized to cDNA using the Thermo Scientific First strand cDNA Synthesis Kit (Thermo Fisher, Waltham, MA, USA) with an RNA template concentration of approx. 500 ng/µL per reaction using random hexamer primers included in the kit.

### 2.3. PCR Amplification and High-Throughput Sequencing

After quantification of total DNA and cDNA with a Quantus Fluorometer (Promega, Germany), the hypervariable V9 region of the 18S rDNA was amplified using the eukaryotic primer set 1389F (5′-TTG TAC ACA CCG CCC-3′) and 1510R (5′-CCT TCY GCA GGT TCA CCT AC-3′) [29] via PCR reaction. PCR mixtures contained 50 ng of total DNA/cDNA template, a final concentration of 0.35 µM for each primer, and VWR Red Taq DNA Polymerase Master Mix (VWR, Germany). The thermal program started with an initial denaturation step at 98 °C for 30 s followed by 25 cycles at 98 °C for 10 s, 57 °C for 30 s, 72 °C for 30 s, and completed with a final elongation step at 72 °C for 10 min. Chimera formation during PCR was reduced by a low number of cycles (25) [30]. To reduce intra-sample variability, PCR reactions were performed in triplicates. Because the results of metabarcoding data strongly depend on the targeted marker region, the hyper-variable V9 region was selected. While being much shorter than the hyper-variable V4 region and less present in public databases, the V9 region represents a good compromise to make a broad diversity of marine taxa visible, but also to recognize some rare species that are neglected when using V4 primers [31].

For subsequent quality measures during data analysis, we created an in vitro community, called a “mock community”, comprising DNA of nine different protist cultures (Table 2) from the HFCC (Heterotrophic Flagellate Culture Collection Cologne). The species were chosen as representatives of the main protist supergroups. DNA of those cultures was isolated using the Quick g-DNA Miniprep kit (Zymo Research, Freiburg, Germany), amplified by PCR (V9 region of the 18S rDNA), purified, and quantified as described for the samples. PCR products of each member of the mock community were then pooled (50 ng of purified PCR product/strain) and added to each individual Next Generation Sequencing run. The Cologne Center for Genomics (CCG, University of Cologne) then performed a paired-end NovaSeq sequencing (2 × 150 bp) run of the amplified fragments. 

### 2.4. Bioinformatic Processing

After sequencing, the raw reads were demultiplexed and processed as follows: barcode and primer sequences were clipped using cutadapt version 2.8 with parameters set to *no-indels*, *m* = 30, and *e* = 0 for the barcodes and *e* = 0.2 for the primer sequences [32]. The next steps were conducted using the dada2 package [33] in R version 4.1.2, starting with the *filter and trim* command and setting the parameters *maxEE* = 1, *truncQ* = 11, *truncLen* = (125, 120), and *maxN* = 0 for quality filtering of the reads. The *errF* and *errR* functions were used to learn the error rates for the dataset. The *derepFastq* function was used for the dereplication of sequences and ASVs were inferred with the *dada* function. The *mergePairs* command merged paired reads with a minimum overlap of 12 nucleotides. As a last quality filtering step, chimeric sequences were removed using the *removeBimeraDenovo* function. By the addition of the V9 region of 150 protist strains from the Heterotrophic Flagellate Collection Cologne, we enlarged the existing PR^2^ database and used it for taxonomic assignment of ASVs via the pairwise alignment function *usearch_global* (version v2.18.0; [34]). Retaining only heterotrophic protist sequences, Metazoa, fungi, autotrophic protists (determined on the basis of taxonomic assignment), as well as unassigned sequences were removed, keeping only ASVs with a pairwise identity of >80% to a reference sequence. As a last filtering step, we used the previously described mock community. Each library preparation was accompanied by one individual mock community, resulting in a total of 18 mock community datasets that were analyzed prior to sample analysis, as described above. For the main dataset of samples, we then chose individual minimum thresholds per sample according to the accompanying mock community on the respective sequencing lane. For calculation of these thresholds, we used the proportion of the lowest read number of an ASV in the mock community data set that could be assigned to the cultured species. ASVs in the sample data sets with a smaller read number than this calculated proportion were discarded. For the 18 accompanying mock communities, the calculated thresholds ranged between 0.02 and 0.07%.

### 2.5. Statistical Analyses

All statistical analyses as well as figures were conducted and plotted with RStudio v2023.03.0. To estimate sequence quality and depth, we calculated rarefaction curves as well as Shannon indices to compare the alpha diversity using the *vegan* package [35]. Non-metric multidimensional scaling (NMDS) analyses were performed to calculate the differences in protist communities between different sediment depths and sampling stations/regions. Therefore, the dissimilarity matrix was calculated based on the Jaccard distance. To compare if those differences were significant, we performed permutational multivariate analyses of variances (PermANOVA) using the *adonis* and *pairwise.adonis* functions. To visualize the proportion of shared and unique ASVs between stations and sediment layers, we used both the R package UpSetR [36] as well as the Treemapify package. To test whether abiotic factors such as salinity, grain size, and water depth had a significant impact on the community composition, we conducted a canonical correspondence analysis (CCA) using the *vegan* package [35] followed by a Monte Carlo permutation test.

The dataset used for the analysis of Fehmarnbelt consisted of six stations with three replicates for the upper 0–2 cm layer and two stations with three replicates for the seven-layer depth profile derived from DNA. Additionally, RNA extractions of samples from the vertical profile of these two stations were analyzed. The dataset for the Oderbank region is smaller, consisting of five stations with five-layer depth profiles (for the two deepest sediment layers, RNA yield was never sufficient for downstream analyses).

## 3. Results

### 3.1. Alpha Diversity of Benthic Protists in the Southern Baltic

**Figure 2:**
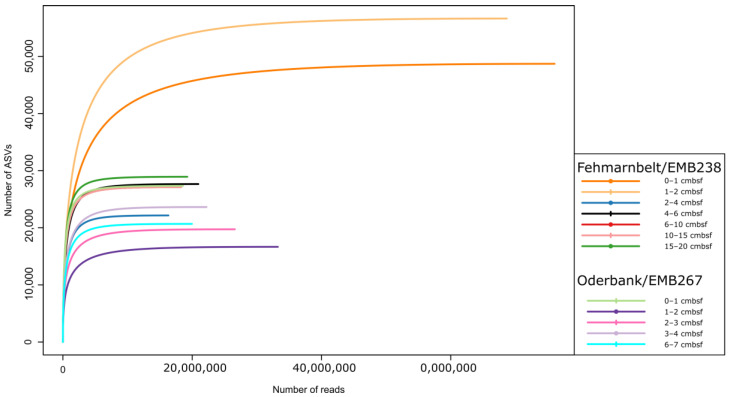
Rarefaction curves of samples from Fehmarnbelt and Oderbank region summed for sediment depth.

After sequencing with NovaSeq, we received data for 129 sediment samples, resulting in a read number of 444,473,336 raw, demultiplexed reads, and 210,074 ASVs for the whole dataset. This results in an average of 3.4 ± 2.9 million reads per sample. Despite the high standard deviation (which was subsequently excluded from analysis), rarefaction curves of all but one sample reached saturation. Summed for sediment depth layers, all curves reached saturation (Figure 2). After the assembling and filtering steps, 293,254,105 reads could be assigned to a sequence from the V9 reference database with a pairwise identity of a minimum of 80%. After the exclusion of Metazoa, fungi, Streptophyta, and exclusively phototrophic taxa, 139,203,557 reads could be assigned to heterotrophic protists. After applying the read threshold derived from the mock community and after manual correction of ambiguous sequences, 78,023,157 reads were clustered into 1233 ASVs. From this dataset, only stations with complete depth profiles were used for further analyses.

**Figure 3:**
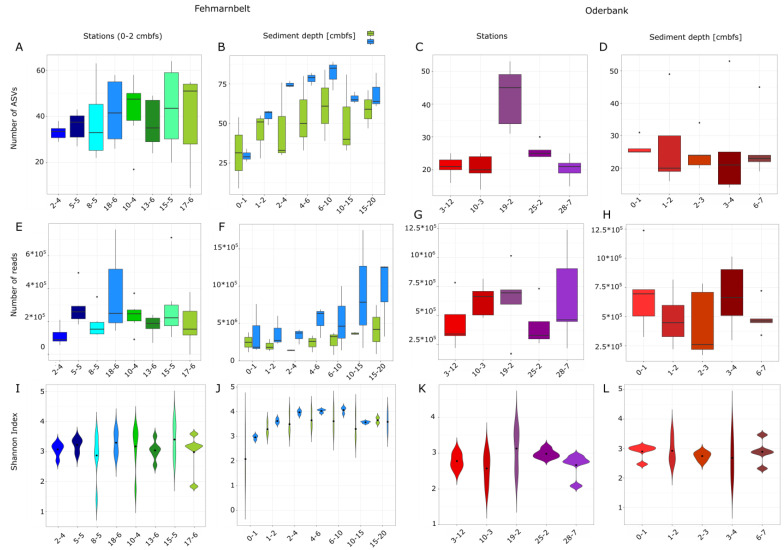
Results of metabarcoding studies of two regions in the western Baltic, Fehmarnbelt, and Oderbank. (**A**) Number of ASVs per station for Fehmarnbelt; (**B**) number of ASVs per sediment depth at Fehmarnbelt; (**C**) number of ASVs per station for Oderbank; (**D**) number of ASVs per sediment depth at Oderbank; (**E**) number of reads per station for Fehmarnbelt and (**G**) for Oderbank; (**F**) number of reads per sediment depth at Fehmarnbelt and (**H**) at Oderbank; (**I**) Shannon index per station at Fehmarnbelt and (**K**) at Oderbank; and (**J**) Shannon index per sediment depth at Fehmarnbelt and (**L**) at Oderbank.

In the Fehmarnbelt region, the uppermost sediment layers (0–2 cm) had an average of 39 ± 13 ASVs assigned to heterotrophic protists and the highest mean number of ASVs was found at station 15-5 with 44 ± 18 ASVs, while the lowest number was found at station 2-4 with 33 ± 3 ASVs (Figure 3). Differences in ASV numbers between the stations regarding these sediment layers were not significant (one-way ANOVA, *p* > 0.5). The Shannon index as an alpha-diversity measure ranged between 2.9 and 3.4, showing no significant difference between the stations (Kruskal–Wallis test, *p* > 0.5) in the upper sediment layers. In the uppermost 2 cm sediment, the highest mean number of reads was detected at station 18-6 with 363,238 ± 254,045 and the lowest at station 2-4 with 111,097 ± 56,751 reads at 0–2 cm sediment depth (Figure 3). Comparing the depth layers (0–1 cm, 1–2 cm, 2–4 cm, 4–6 cm, 6–10 cm, 10–15 cm, and 15–20 cm) of cores for stations 17-6 and 18-6, the highest mean number of ASVs was found at 6–10 cm sediment depth for both stations with 61 ± 23 ASVs at station 17-6 and 82 ± 9 ASVs at station 18-6 (Figure 3). The Shannon index for the different layers ranged between 2.5 and 4.1 (Figure 3), the differences were found to be significant (Kruskal–Wallis test, *p* < 0.05).

The highest mean number of reads was found for station 17-6 with 417,669 ± 464,385 (high standard deviation results from one sample with only 89,299 reads) at 15–20 cm sediment depth and the lowest number of reads was found for station 17-6 in the 2–4 cm sediment layer with 137,696 ± 8444 ASVs (Figure 3). For station 18-6, the highest mean number of reads was detected at 15–20 cm with 947,200 ± 540,776 ASVs and the lowest at 2–4 cm sediment depth with 333,787 ± 104,046 ASVs.

For station 17-6 from Fehmarnbelt, we found that for RNA, the mean ASV number was 54 ± 11, and for station 18-6, 46 ± 18. For RNA at station 18-6, the mean read numbers were 364,111 ± 349,887, and for station 17-6, 340,954 ± 184,354. 

In the Oderbank region, the stations had an average number of 26 ± 10 ASVs, the highest number of ASVs was detected at station 19-2 with 42 ± 9 ASVs, and the lowest value at station 10-3 with 20 ± 4 ASVs. The differences in these numbers were not significant (Kruskal–Wallis test, *p* > 0.5). The Shannon index between the stations ranged between 2.0 and 3.6 but showed no significant differences (one-way ANOVA, *p* > 0.5). The lowest mean number of reads was detected at station 25-5 with 413,163 ± 191,111 reads, and the highest at station 19-2 with 639,953 ± 304,727 reads (Figure 3).

Looking at the different depth layers in the Oderbank region (0–1 cm, 1–2 cm, 2–3 cm, 3–4 cm, 6–7 cm) the lowest mean number of ASVs was found in layer 2–3 cm with 25 ± 6 ASVs, and the highest in layer 6–7 cm with 27 ± 10, as well as in 1–2 cm with 27 ± 13 ASVs. The differences in the numbers were significant (Kruskal–Wallis test, *p* < 0.05). The Shannon index ranged between 2.0 and 3.6 and was found to not be significantly different (one-way ANOVA, *p* > 0.05). The lowest mean number of reads could be detected at 2–3 cm sediment depth with 428,497 ± 292,736 and the highest at 0–1 cm with 699,095 ± 342,518 reads (Figure 3).

### 3.2. Protist Community Composition at Different Regions and Sediment Depths 

**Figure 4:**
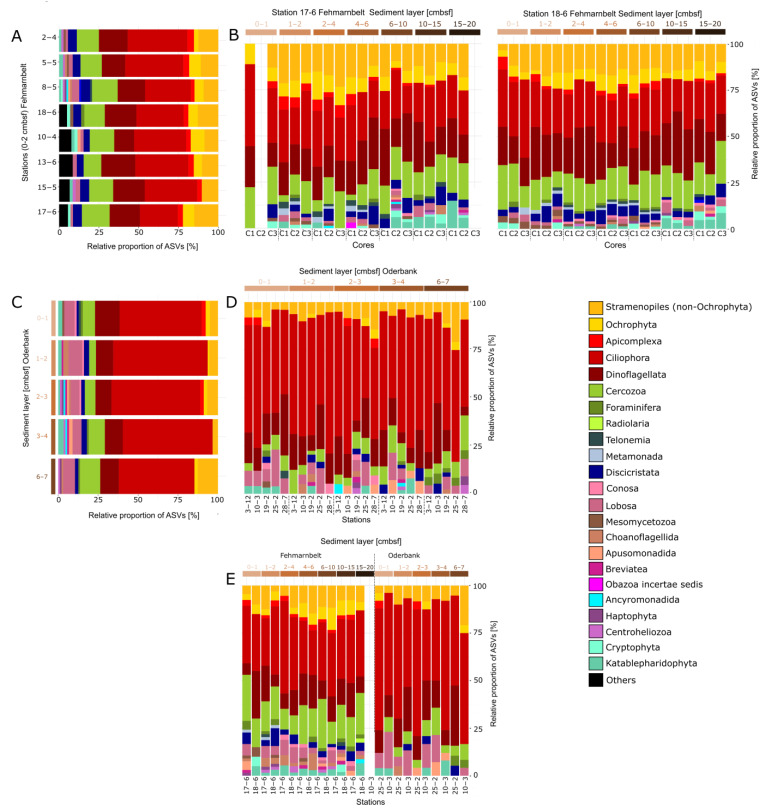
Community composition of benthic protists at Fehmarnbelt and Oderbank showing the relative proportion of ASVs assigned to taxonomic groups. (**A**) Comparison of all samples from 0–2 cmbsf at Fehmarnbelt; (**B**) protist community structure obtained from depth profiles of cores (7 different depths, each with 3 replicates) at stations 17-6 and 18-6 at Fehmarnbelt; (**C**) vertical distribution of the protist community structure at Oderbank (summed for all stations from 5 different depth layers); (**D**) vertical changes in community structure for the different stations at Oderbank; and (**E**) direct comparison of the protist community structure of two stations from both regions, Fehmarnbelt and Oderbank, regarding different sediment layers based on RNA ASVs.

The data for the uppermost 2 cm of sediment at all stations of the Fehmarnbelt were dominated by Ciliophora (Figure 4A) with relative proportions of ASVs between 24% (station 17-6) and 38% (station 2-4), followed by Dinoflagellata with 13% (station 10-4) up to 21% (station 13-6), followed by Cercozoa with relative proportions between 11% (station 13-6) and 17% (station 17-6). The largest proportions of ciliate ASVs belong to the Litostomatea (18%), Spirotrichea (18%), and Oligohymenophorea (20%). Among the Dinoflagellata, almost 80% of taxa belong to the Dinophyceae, and among the Cercozoa, most belong to the Filosa-Thecofilosea (55%).

The depth profiles (seven layers) for all three cores of stations 17-6 and 18-6 in Fehmarnbelt were compared. At station 17-6, almost all layers were again dominated by ASVs belonging to Ciliophora with 23% to 44% of relative proportions of ASVs, followed by either Stramenopiles (non-Ochrophyta) with 20% to 23%, Cercozoa with 18–20%, or Dinoflagellata with 17–24% of ASVs. Much lower proportions were reached by Katablepharidophyta, mainly in the lower sediment layers (3–15%, Figure 4B). The largest proportion of ciliate taxa belonged to the Litostomatea (20%), Labyrinthulea were most abundant among Stramenopiles (38%), and Dinophyceae dominated the Dinoflagellata (80%). 

A similar pattern was obtained for station 18-6. The most dominant groups of ASVs belonged to the Ciliophora (17–42%, with Oligohymenophorea and Spirotrichea both 19%), Dinoflagellata (13–33%, with 80% Dinophyceae), Cercozoa (8–24%, 70% of which belong to Filosa-Thecofilosea) and Stramenopiles (non-Ochrophyta) with 3–18% (49% of which belong to Labyrinthulea). Again, a rise of Katablepharidopyhta taxa was observed towards deeper sediment layers. They contributed 5–9% of ASVs in the deepest layer 15–20 cm (vs. ~1% in the upper layers; Figure 4B).

In the Oderbank region, Ciliophora taxa were even more dominant in all sediment layers compared to the Fehmarnbelt. With relative proportions between 38 and 74%, they made up a large proportion of the whole community (Figure 4C,D). The largest proportion (20%) of ASVs belonged to Spirotrichea, followed by Karyorelicta (18%). The second largest relative proportion of ASVs was contributed by Dinoflagellata with 6–32% (with 70% belonging to the Dinophyceae), followed by Stramenopiles (5–21%, of which 30% belong to the MAST groups and 30% to bicosoecids) and Cercozoa (2–18%, with 62% belonging to the group of Filosa-Thecofilosea). Apart from that, no clear pattern of taxa distribution in relation to sediment depth was visible. Summing up ASVs of all stations sorted for sediment depth, one group (Telonemia) was only present in the uppermost layer, whereas the taxa belonging to the Katablepharidophyta were absent in the deepest layer (6–7 cm), while Cercozoa were present to a larger proportion in the deepest layer.

Apart from these minor differences in community composition, both regions did not show remarkable differences in the vertical distribution of protists when only large taxonomic groups are considered. As we could only obtain RNA data for two stations of the Fehmarnbelt region (stations 17-6 and 18-6, Figure 4E), we randomly chose two stations of the Oderbank region (stations 10-3 and 25-2) for direct comparison. While Fehmarnbelt samples were dominated by several groups in more similar proportions (Ciliophora 24–49%, Dinoflagellata 6–25%, Cercozoa 10–26%), Oderbank was highly dominated by Ciliophora (up to 74%). Samples from Fehmarnbelt seem to show a larger variety of taxonomic groups (22, compared to 12 at Oderbank). In this sense, a taxonomic group represents the taxonomic rank of division, above the class rank.

### 3.3. Protist Beta-Diversity in Relation to Sediment Depth

**Figure 5:**
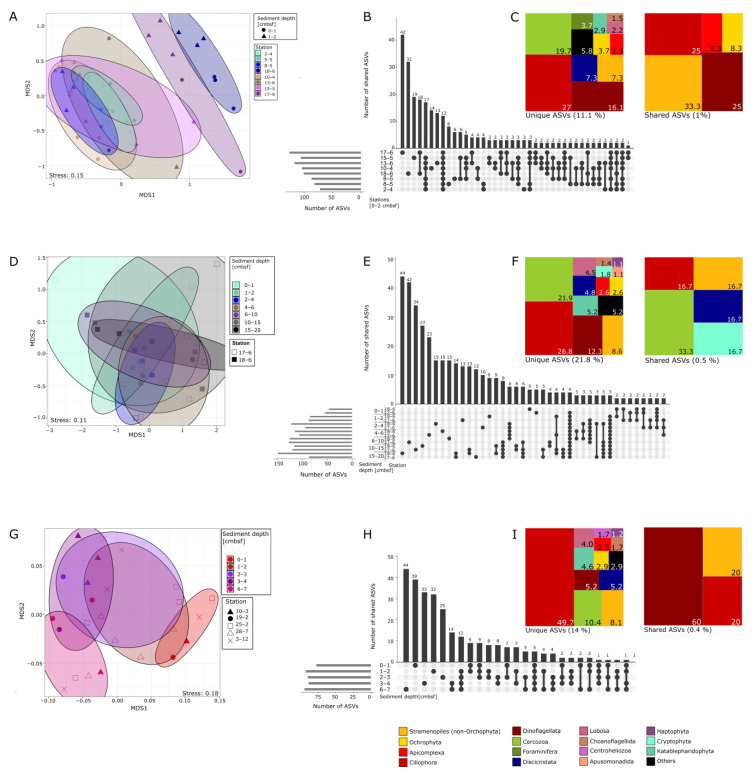
NMDS plot based on the Jaccard Index comparing benthic protist communities of the different stations in the western Baltic. (**A**) NMDS plot comparing all stations at Fehmarnbelt for the surface sediment layer 0–2 cmbsf. (**B**) Upset plot showing the number of shared ASVs between the different Fehmarnbelt stations (top bar chart) or unique to one station, as well as the overall number of ASVs (horizontal bars). Connected dots below the bar chart mean ASVs are shared between two or more stations. (**C**) Tree map showing the relative proportion of shared and unique ASVs per taxa group at Fehmarnbelt stations for the 0-2 cmbsf sediment layer. (**D**–**F**) Comparison of the vertical distribution of ASVs of the two Fehmarnbelt stations 17-6 and 18-6 in a similar manner as for (**A**–**C**). (**G**–**I**) Comparison of the community structure regarding all different sediment layers from stations at Oderbank analyzed in a similar manner as for (**A**–**C**).

NMDS analyses revealed a higher resolution of taxonomic composition regarding sampling stations and sediment depth. In the 0–2 cm layer of the Fehmarnbelt samples, the protist communities formed significantly separate clusters regarding the different stations (permANOVA, *p* = 0.001, Figure 5A).

Stations 17-6 and 18-6, in particular, cluster quite separately, with almost no overlap with the other stations (Figure 5A). This phenomenon is still visible when the depth profiles of stations 17-6 and 18-6 are compared, where no significant differences between sediment depth but between the two stations were recorded (permANOVA, *p* < 0.01, Figure 5D,E). In the uppermost sediment layers, we found the highest number of unique ASVs at station 17-6 with a relative proportion of 16%, followed by station 18-6, with a relative proportion of 12%. Overall, the stations only shared 1% of ASVs, divided between the most dominant groups, with ASVs of Stramenopiles (non-Ochrophyta) at 33.3% and Ciliophora as well as Dinoflagellata both at 25%. With regard to the depth layers, the highest numbers of unique ASVs were detected in the deeper sediment layers, with 11% of unique ASVs found in 15–20 cm depth at station 18-6 followed by station 17-6 in 6–10 cm sediment depth, also with 11%. Overall, the two stations shared only 0.5% ASVs, with cercozoans being the most dominant group (33.3%).

For the Oderbank region, the NMDS analysis showed significant differences in community composition between the sediment layers (permANOVA, *p* = 0.001, Figure 5G), but not between the stations within the region. Therefore, the layers were summed up for all stations for comparison. With 16%, the highest number of unique ASVs was found in 6–7 cm depth, directly followed by 14% of unique ASVs in 0–1 cm (Figure 5H), explaining the significant differences between the layers. While the layers overall only shared 0.4% of ASVs, most taxa were shared out of the clade of Dinoflagellata at 60% (Figure 5I).

**Figure 6:**
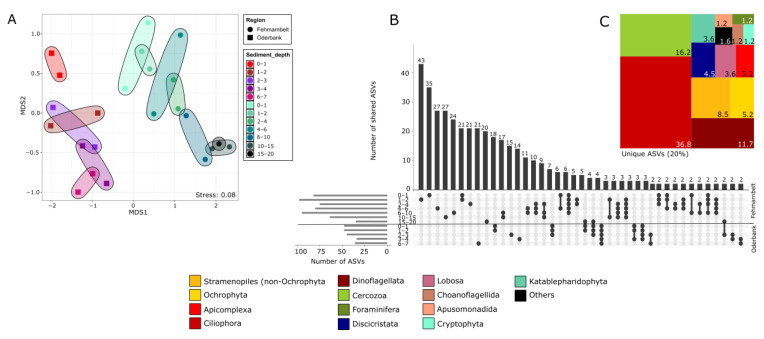
NMDS plot based on the Jaccard distance comparing protist communities in different sediment depth layers of two sampling stations in the Oderbank region and two in the Fehmarnbelt region (**A**). (**B**) Upset plot showing the number of shared or unique ASVs for the different sampling depths for both regions (bar chart at the top). Connected dots below each bar show shared ASVs between different depths and stations. Horizontal bars indicate the total number of ASVs for the two stations in each region. (**C**) Tree map showing relative proportions of ASVs for taxa groups unique to one depth and region.

The NMDS analysis revealed a clear separation of the compared protist communities from the two stations of Oderbank (stations 10-3 and 25-2) and Fehmarnbelt (stations 17-6 and 18-6) based on RNA-derived data (permANOVA, *p* = 0.001, Figure 6). On the basis of the rigid filtering of the data set using the mock community, the two chosen stations from Oderbank shared no ASVs with the two stations of Fehmarnbelt (Figure 6B,C). They instead displayed a high percentage of unique ASVs that were not shared between all 12 sediment layers (Figure 6B). The highest number of unique ASVs for Oderbank and Fehmarnbelt was found among the Ciliophora group.

## 4. Discussion

Even though metabarcoding studies of protist communities have become much more frequent in the past 15 years, the majority of studies still concentrate on pelagic protist communities [3]. While studies on benthic communities are scarce, benthic brackish water communities are even more poorly studied and metabarcoding studies of benthic protists of the Baltic Sea are basically non-existent. According to our knowledge, our study represents the first metabarcoding approach to estimate the benthic protist community of sediments in the Baltic Sea and aims to better understand their biodiversity and ecological roles. By targeting the V9 region of the 18S rDNA, we chose a suitable region to estimate the overall richness of the protist community in the Baltic Sea, also including rare taxa [31]. As mentioned above, benthic protist communities for the Baltic Sea have so far received relatively little attention. It is therefore likely that Baltic Sea-specific members of the protist community are underrepresented in the reference databases. To verify and improve the outcome of the analysis, we chose to add an additional filtering step using a mock community. The addition of a mock community as a supplementary sample in a next-generation sequencing run has been recommended by several studies [37,38,39], especially as a measure to eliminate “noisy” sequences. We adapted those ideas to create individual read thresholds for each library preparation. The rather strict limit values derived in this way served as an additional form of quality control. It has been shown that the overall impression of the community composition does not dramatically change when these thresholds are applied [39]. On the other hand, applying a strict filter increases the likelihood that differences in species composition between stations will be overemphasized. This could be the reason for the relatively high level of uniqueness we found for many species.

As no previous metabarcoding data on benthic protist communities in the Baltic Sea seem to exist, it is hard to compare our results on the basis of molecular data sets. Additionally, the specific nature of the brackish water environment allows only limited comparisons to studies from either marine or freshwater environments, and different bioinformatic pipelines may additionally influence the results. There are only a few quantitative and qualitative studies from the regions based on direct counts using light microscopy. Benthic ciliates were intensively studied at a station in the Kiel Bight [17], in the vicinity of the Fehmarnbelt stations; however, the water depth of the region studied by Sich [17] was much shallower and sandier than the region investigated in the present study. In another study of benthic ciliates in the vicinity of the Oderbank region [22], again the sampling site was shallow, though similar in the sediment quality. In both cited studies, karyorelictid, spirotrich, litostome, and oligohymenophoreans were dominant, comparable to the present investigation using molecular techniques. Regarding benthic flagellated protists, only the shallow-water study near Ruegen Island [22] was available for comparison. The comparison with our metabarcoding studies shows that flagellate groups recorded from live counting were also recovered by the metabarcoding studies.

Regarding the community composition obtained by our metabarcoding study, Ciliophora were the main dominant group regarding the number of ASVs in Baltic Sea sediments (with differing proportions regarding the region), but on the sides of read abundances, the MALV-I clade, a rather poorly studied group of marine Syndiniales with only a few cultured species [24], reached by far the highest read abundances. Sequences of the MALV group are known to dominate in DNA studies, which is, most plausibly, because they have higher rDNA copy numbers [40] and may not reflect actual activity. Still, also in the dataset from Oderbank derived only from RNA, an ASV representing a sequence from the MALV-I 4 group has the highest read abundance. In line with previous studies [24], the largest proportion of ASVs of the MALV clade in the dataset belonged to the MALV-1 group known to be predominant in anoxic environments and hydrothermal vents, and seems to be common in sediments.

**Figure 7:**
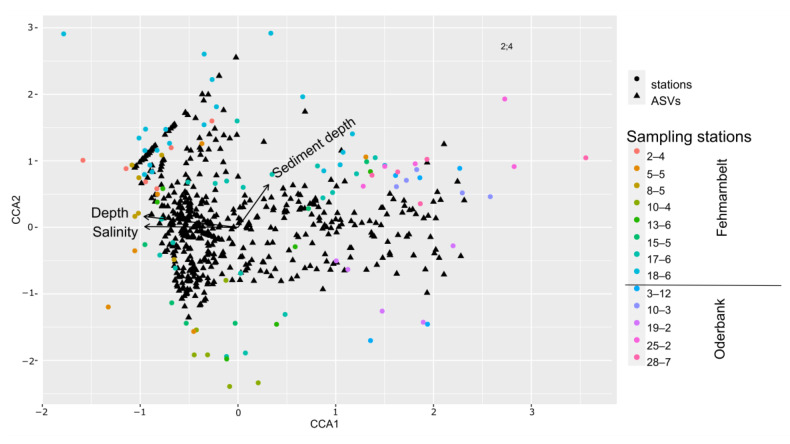
CCA analysis including a comparison of the benthic protist community composition based on ASVs including all stations of Fehmarnbelt and Oderbank regions analyzing the influence of water depth, sediment depth, and salinity.

Benthic ciliates are known to have a high species richness in brackish water environments [41], especially in the Baltic Sea [42], proposing that salinity can have a negative effect on species richness. It is therefore not surprising that our analyses showed a high proportion of ASVs belonging to ciliates. Similar patterns have been found for planktonic organisms in the Baltic Sea [43], refuting the theory that the taxonomic diversity of organisms is lowest in the horohalinicum [44]. A CCA of the complete dataset has shown that out of several abiotic factors, salinity had a significant effect on the benthic protist community (Monte Carlo permutation, *p* = 0.001; Figure 7) as well as water depth (*p* = 0.003) and sediment depth (*p* = 0.002). For a Pacific littoral region, Gong et al. [45] showed that water depth had the strongest influence on α- and β-diversity of benthic protist communities.

**Figure 8:**
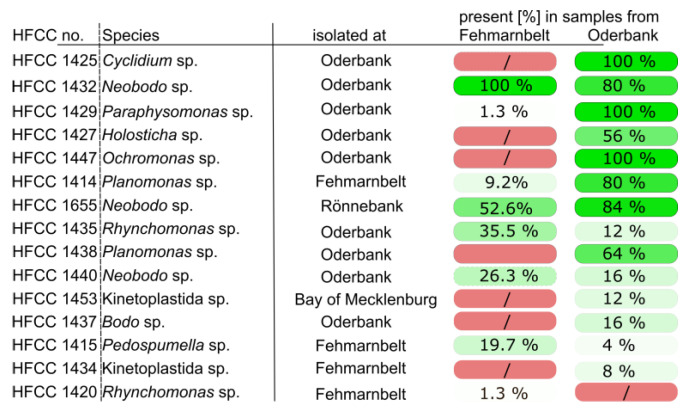
List of genotypes obtained from species that were cultivated from samples collected from the two investigated regions. HFCC stands for the number of the Heterotrophic Flagellate Culture Collection Cologne. The red labeled data indicate that the genotype could not be recovered in metabarcoding studies of the respective region. The number shows the percentage of samples in which they were detected in a regional dataset.

Apart from salinity, grain size not only has an effect on functional ciliate diversity—implying that coarser sediment promotes free-swimming species with an elongated cell form, whereas fine sediment houses species with crawling behavior and flattened cell bodies [41]—but also on the abundance of ciliates, which was shown to be positively correlated to median sediment grain size [46,47]. In contrast to these studies, our results obtained from CCA analysis could not verify that grain size has a significant influence on the protist community as a whole. At Fehmarnbelt, a region with approx. 19 PSU salinity and median grain size of approx. 55 µm, the relative proportion of ASVs belonging to ciliate taxa make up about 44% of the overall number of ASVs, comprising 100 different ASVs of ciliate taxa in total. At Oderbank, we measured a salinity of about 8 PSU and a median grain size of 178 µm. Here, we found a much higher relative proportion of ASVs belonging to ciliates of up to 78%, with 143 different ASVs. Still, one has to keep in mind that the majority of samples from Fehmarnbelt were derived from DNA studies while that from Oderbank originate from RNA. Sediments can act as storages of DNA sunken down from the water column and therefore might contain also DNA of pelagic species, which might lead to an overestimation of diversity [48]. However, most of the dominant ciliate ASVs, for instance, belonged to well-known benthic taxa. Several protist species are known to have remarkably high abilities to adapt to different salinities. We showed that among other Stramenopiles of the genus *Cafeteria*, *Cafeteria baltica*, isolated from sediment of the Fehmarnbelt, can tolerate salinities between 0 and 125 PSU [49]. We, therefore, assume that at least some protist species are ubiquitously dispersed in the Baltic Sea, independent of the salinity. This assumption is supported by the fact that we were able to retrieve several protists from an accompanying cultivation approach that were also recovered from the dataset of Oderbank and Fehmarnbelt (Figure 8).

Of course, the results of cultivation approaches may be biased by the fact that mostly generalists or especially robust organisms are easier to cultivate and therefore do not reflect the actual diversity. Nevertheless, it shows that several protist strains isolated from the two study regions are able to live under various abiotic conditions in the laboratory, and, importantly, the recovery of sequences of cultivated protists from the respective region verifies our metabarcoding study.

A large proportion of ASVs, both from Fehmarnbelt and Oderbank, was assigned to the Stramenopiles and Cercozoa. At Fehmarnbelt, the highest proportion of ASVs belonging to the Stramenopiles was assigned to Labyrinthulea, a class of Stramenopiles known mainly from marine and estuarine environments [50]. Labyrinthulids are able to decompose marine detritus by extracellular hydrolytic enzymes [5]. In their role as decomposers, they are typical inhabitants of sediments that are rich in organics [51] and could be typical for the eutrophic environment of the Baltic Sea. At Oderbank, stramenopile sequences mainly belonged to bicosoecids and the MAST group. While the bicosoecids detected in the samples from Oderbank could not be assigned to a level lower than the class level, it is hard to make any specific comments. Sequences of the MAST group (Marine Straminopiles) belonged to different ribogroups regarding their phylogenetic position, but also according to their ecological preferences [25]. At Oderbank, 50% of the ASVs were assigned to the MAST-2 group, which comprises mainly marine—but also some freshwater—species originating from different geographic regions. This group is known to be exclusively planktonic and mainly occurs in oxic (sometimes also micro-oxic) environments [25]. At Oderbank, ASVs belonging to this group were found in the surface sediment layer, but also in deeper layers (maybe originating from encysted cells). Surprisingly, not one of the ASVs showed 100% identity to the sequences deposited in the reference database (highest identity of 99.2%), this underlines that the Baltic Sea is under-sampled and therefore underrepresented in public databases.

Compared to a metabarcoding study, which investigated the diversity of pico- up to mesoplankton in the Baltic Sea along a salinity gradient [16], there are some similarities to the Arkona Sea, which was their planktonic sampling site closest to our sampling region in the Oderbank. The major taxa groups were composed of similar classes of organisms to the ones in our studies of the sediment, which underlines the idea that the sediment might act as a sink for the planktonic diversity. An example is that, e.g., *Strombidium*, an oligotrich ciliate, was found with high dominance in the planktonic samples, and was also present with high read abundances in Fehmarnbelt sediments. High read numbers of the MAST-2 group occurred in plankton samples and were also found, especially in Oderbank samples.

Another important factor shaping protist communities is the availability of oxygen. While some protist taxa are able to survive both under aerobic and anaerobic conditions, others are sensitive to either one or the other condition [8,52]. Anaerobic ciliates are known to possess certain organelles, called hydrogenosomes, to ferment pyruvate into acetate and H_2_ [53]. The protist community in the oxidized surface layers of the sediment was found to be different from the deeper sediment layers. While the exact O_2_ content of the sediment layers was not measured during our study, we observed dark spots in the sediment layers, indicating anaerobic conditions already at 2 cmbsf. Even though the community of the different depth layers at Fehmarnbelt did not show a significantly different community, a high number of unique and unshared ASVs were found, especially in the deeper sediment layers. For Oderbank, we could detect significant differences, even though the sediment layers did not go as deep as those of Fehmarnbelt. At Oderbank, we could detect ciliate species known to be able to survive anaerobic conditions ([53]; e.g., *Trimyema*, *Lacrymaria*, *Caenomorpha*) in almost all sediment layers, indicating, at least, anaerobic patches in the sediment. For Fehmarnbelt, we could detect *Trimyema*, *Metopus,* and also *Lacrymaria* in many layers of the sediment. Besides salinity and water depth, the CCA of the complete dataset showed a significant influence on the sediment depth on the community composition (*p* = 0.002, Figure 7).

As part of the microbial food web, the abundance of protists is closely linked to the predominant bacterial community and abundance [22,54], which are also heavily influenced by abiotic factors and sediment properties [55]. Therefore, it is very likely that the bacterial community in both regions differ, thereby substantially affecting the protist community as well, and vice versa. Studies on prokaryotes are carried out at the moment and might reveal interesting data for comparative analyses in the future.

To analyze if there are differences in community composition between Fehmarnbelt and Oderbank, we compared the RNA-derived dataset of two stations from Fehmarnbelt with two randomly chosen stations at Oderbank. At least for those four stations, we could show that the protist communities form two distinct clusters for the two regions with no shared ASVs, at least not when we use our strict filtering step. Still, we know from our cultivation approach that there are at least a few taxa that appear in both datasets. In terms of the distribution and diversity of protists, several partly contradicting hypotheses have been established during the last years [56] that also addressed the main problems of estimating protist diversity, which includes under-sampling. Other studies have shown that the seafloor can be very heterogeneous regarding protist diversity even at a small spatial scale [57]. More data are needed to draw robust conclusions regarding the differences and similarities of benthic protist communities in the Baltic Sea. Nevertheless, our study might give novel insights into protist diversity for the vastly understudied benthic protist community of the Baltic Sea.

## 5. Conclusions

Our study on the community of benthic protists in the Baltic Sea obtained via metabarcoding of the V9 region of 18S rDNA showed significant differences in community composition not only between the different sampling regions but also between different sediment layers. For both regions, ASVs belonging to Ciliophora dominated the overall community, especially at Oderbank. Dinoflagellata, Stramenopiles, and Cercozoa showed also high diversity, but differed with regard to the lower taxonomic groups between the two regions. We assume that certain abiotic factors such as salinity, sediment grain size, and availability of oxygen are responsible for the differences in the communities, even though there are some taxa being ubiquitously distributed in both regions.

## Figures and Tables

**Table 1:** List of sampling stations relevant to this study. Sampling region (FB = Fehmarnbelt, OB = Oderbank), station/cast in the region, area (MPA = marine protected area, Ref. area = reference area), and the GPS position of the stations are given. The depth intervals at which MUC cores were cut are indicated (cmbsf = cm below seafloor), and cruise number and sediment type at the stations are added.

Region	Station/Cast	Area	Longitude/Latitude	Depth Intervals [cmbsf]	Depth [m]	Cruise	Sediment Type
FB	2-4	MPA	54°33.37′ 10°45.52′	0–1, 1–2, 2–4, 4–6, 6–10,10–15,15–20	23.5	EMB238	muddy
FB	5-5	MPA	54°32.77′ 10°46.61′	0–1, 1–2, 2–4, 4–6, 6–10,10–15,15–20	23	EMB238	muddy
FB	8-5	MPA	54°33.08′ 10°45.63′	0–1, 1–2, 2–4, 4–6, 6–10,10–15,15–20	23.9	EMB238	muddy
FB	10-4	Ref. area	54°32.36′ 10°43.49′	0–1, 1–2, 2–4, 4–6, 6–10,10–15,15–20	22.8	EMB238	muddy
FB	13-6	Ref. area	54°32.34′ 10°43.55′	0–1, 1–2, 2–4, 4–6, 6–10,10–15,15–20	23	EMB238	muddy
FB	15-5	Ref. area	54°32.51′ 10°41.71′	0–1, 1–2, 2–4, 4–6, 6–10,10–15,15–20	23.2	EMB238	muddy
FB	17-6	Ref. area	54°32.5′ 10°41.16′	0–1, 1–2, 2–4, 4–6, 6–10,10–15,15–20	23	EMB238	muddy
FB	18-6	MPA	54°32.93′ 10°46.11′	0–1, 1–2, 2–4, 4–6, 6–10,10–15,15–20	24.4	EMB238	muddy
OB	3-12	MPA	54°15.774′ 14°19.148′	0–1, 1–2, 2–3, 3–4, 6–7, 8–9, 10–11	15.3	EMB267	sandy
OB	10-3	MPA	54°15.438′ 14°19.733′	0–1, 1–2, 2–3, 3–4, 6–7, 9–10, 10–15	14.9	EMB267	sandy
OB	19-2	Ref. area	54°14.934′ 14°18.435′	0–1, 1–2, 2–3, 3–4, 6–7, 9–10, 14–15	15.5	EMB267	sandy
OB	25-2	Ref. area	54°15.655′ 14°16.873′	0–1, 1–2, 2–3, 3–4, 6–7, 9–10, 13.5–14.5	15.9	EMB267	sandy
OB	28-7	Ref. area	54°15.406′ 14°17.241′	0–1, 1–2, 2–3, 3–4, 6–7, 9–10, 14–15	15.5	EMB267	sandy

**Table 2:** List of organisms used for the “mock community”.

HFCC No.	Species	Protist Group
171	Rhynchomonadidae undet.	Kinetoplastida
175	*Fabomonas tropica*	Ancyromonadida
176	*Massisteria marina*	Cercozoa
178	*Ministeria vibrans*	Opisthokonta
203	*Cafeteria burkhardae*	Stramenopiles
744	*Aristerostoma* sp.	Ciliophora
766	*Protocruzia* sp.	Ciliophora
768	*Halocafeteria* sp.	Stramenopiles
828	*Neobodo* sp.	Kinetoplastida

## Data Availability

The data presented in this study are available on request from the corresponding author. At the time of publication of this study, the data were submitted to PANGAEA and will be available soon.
